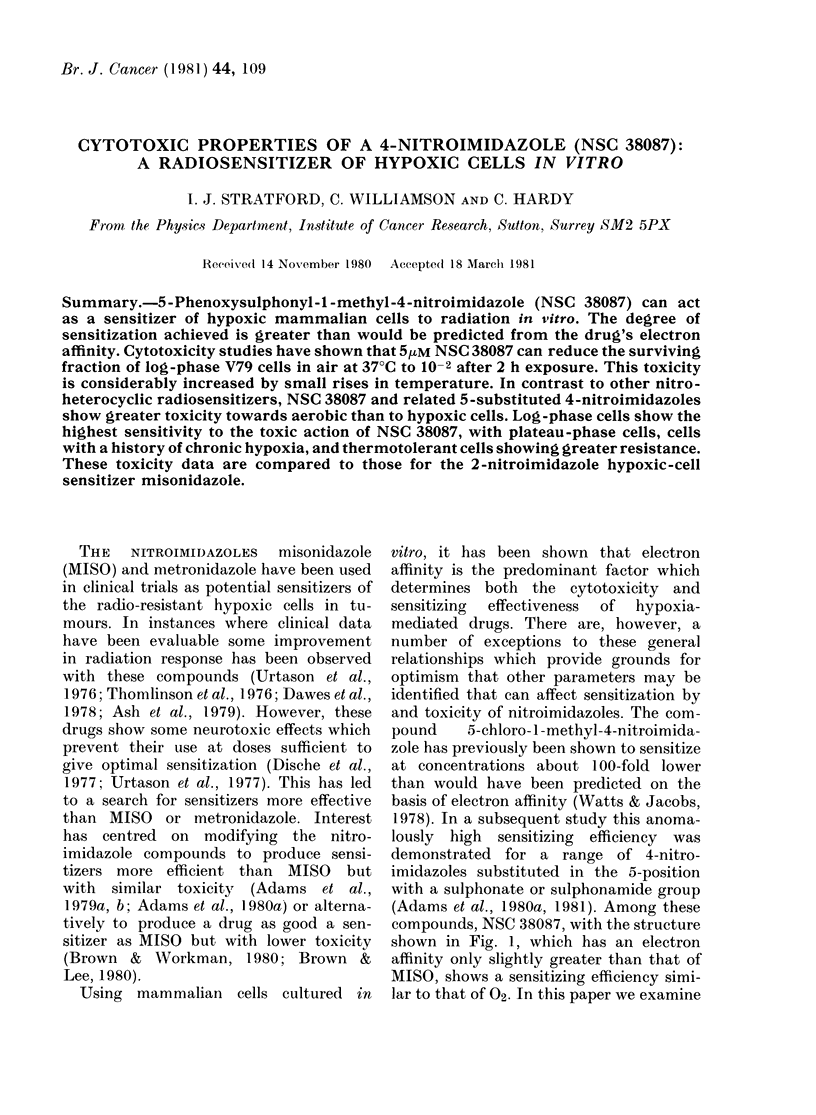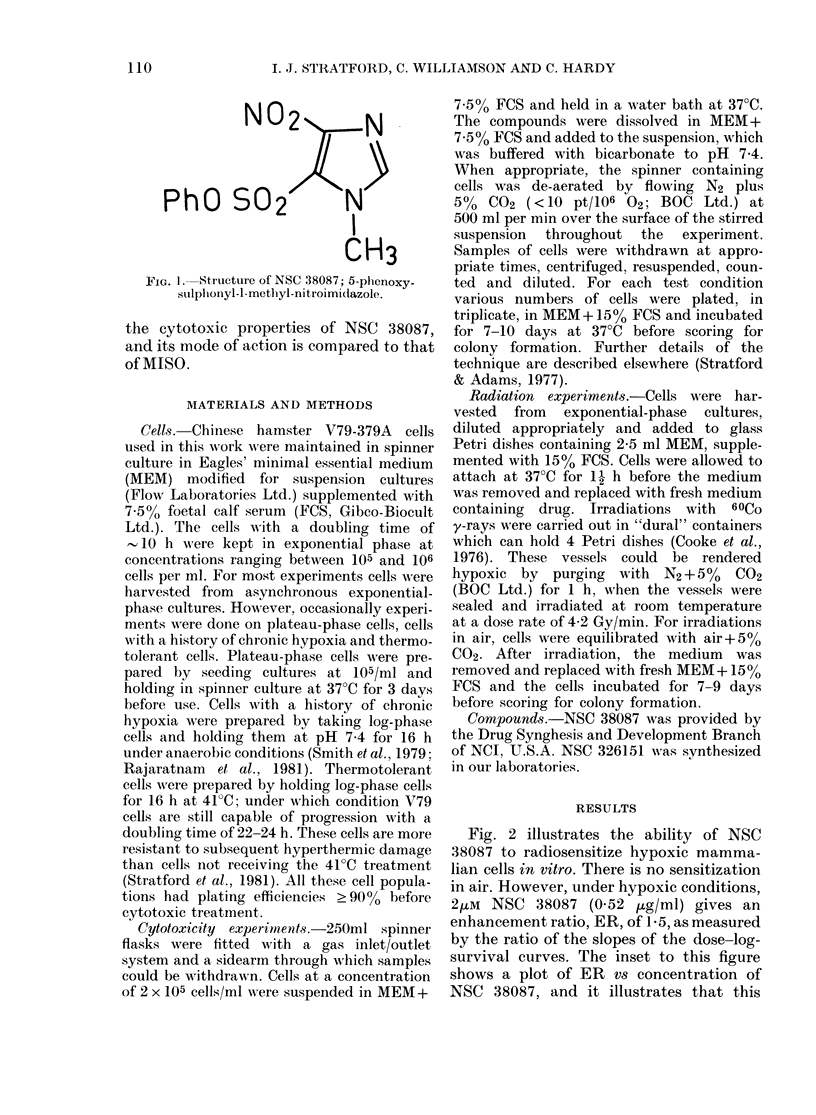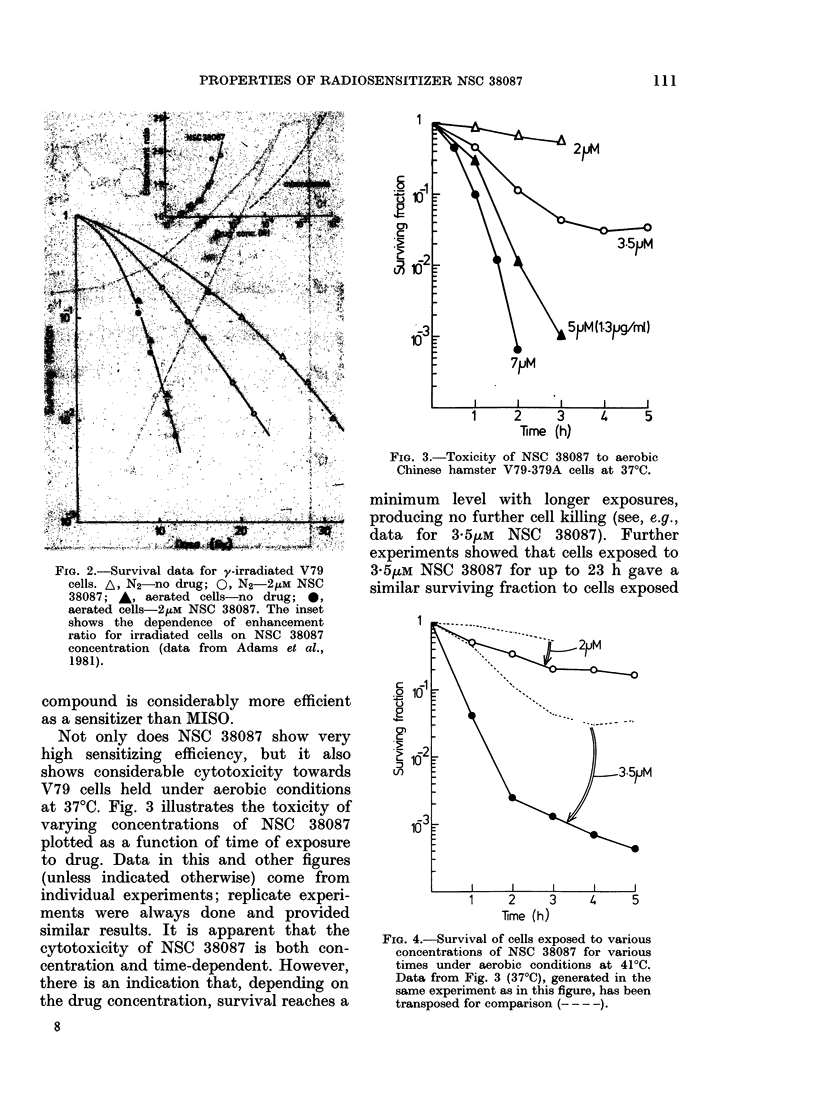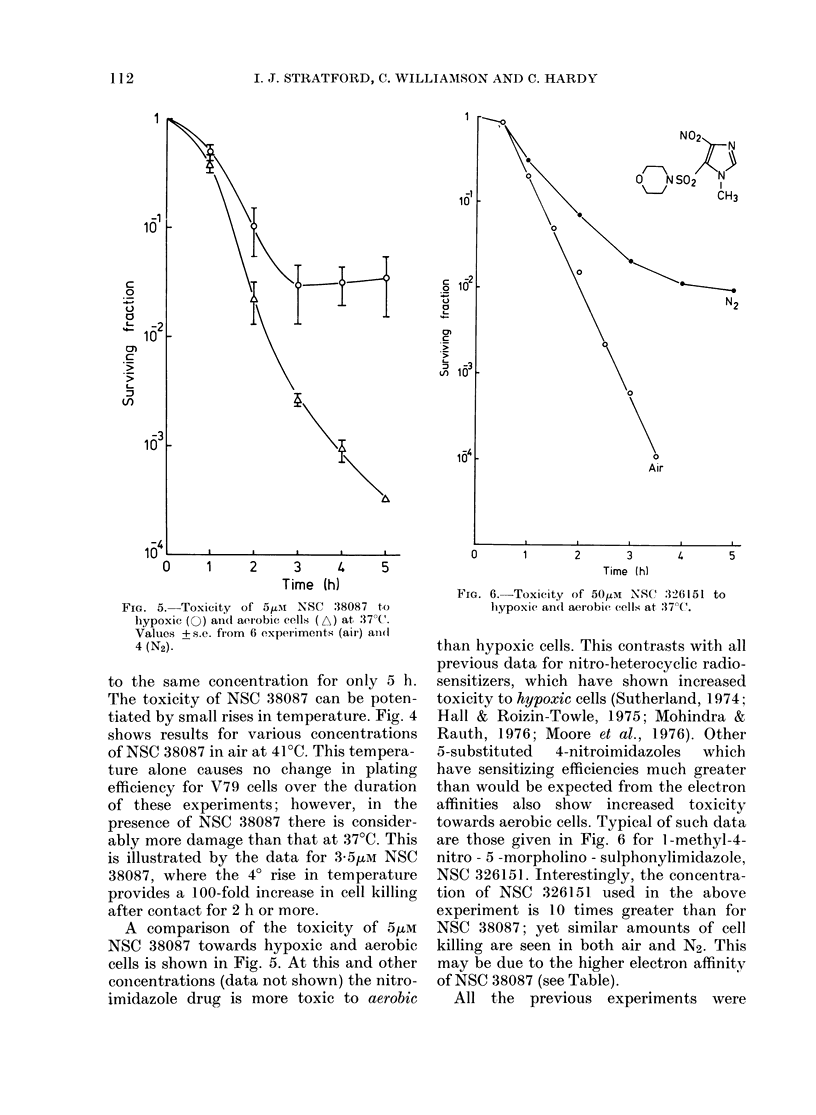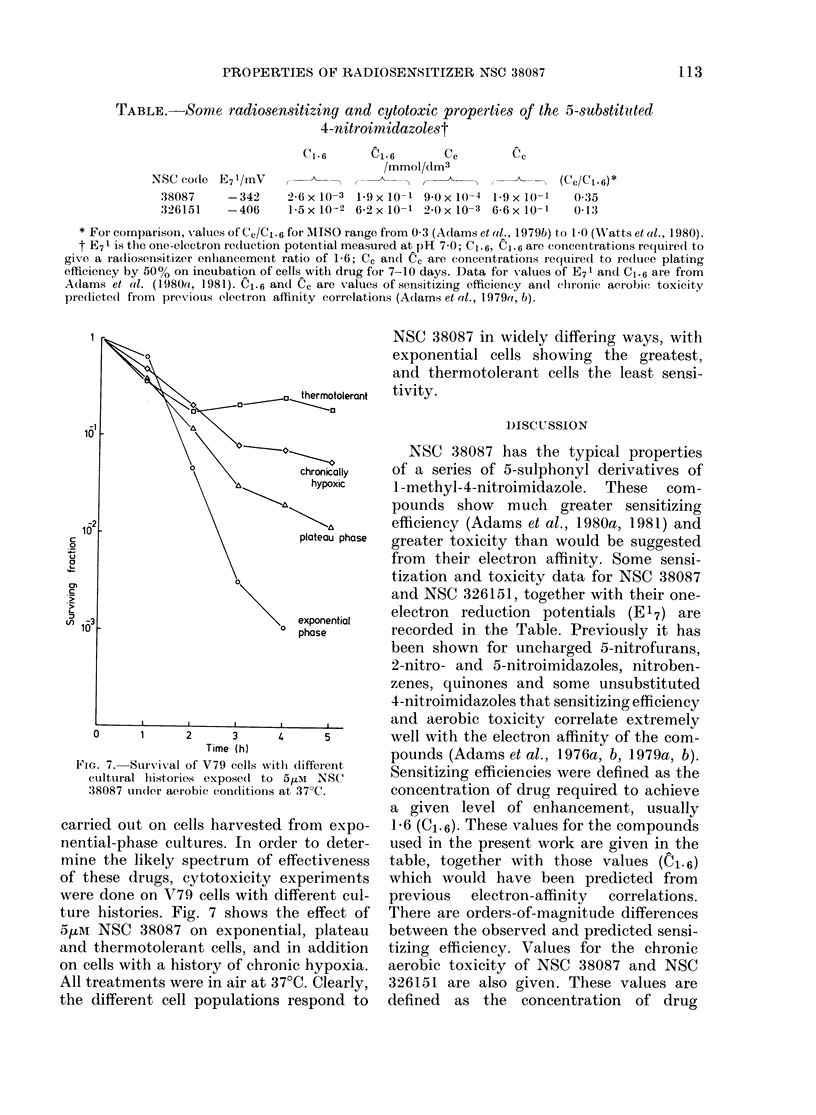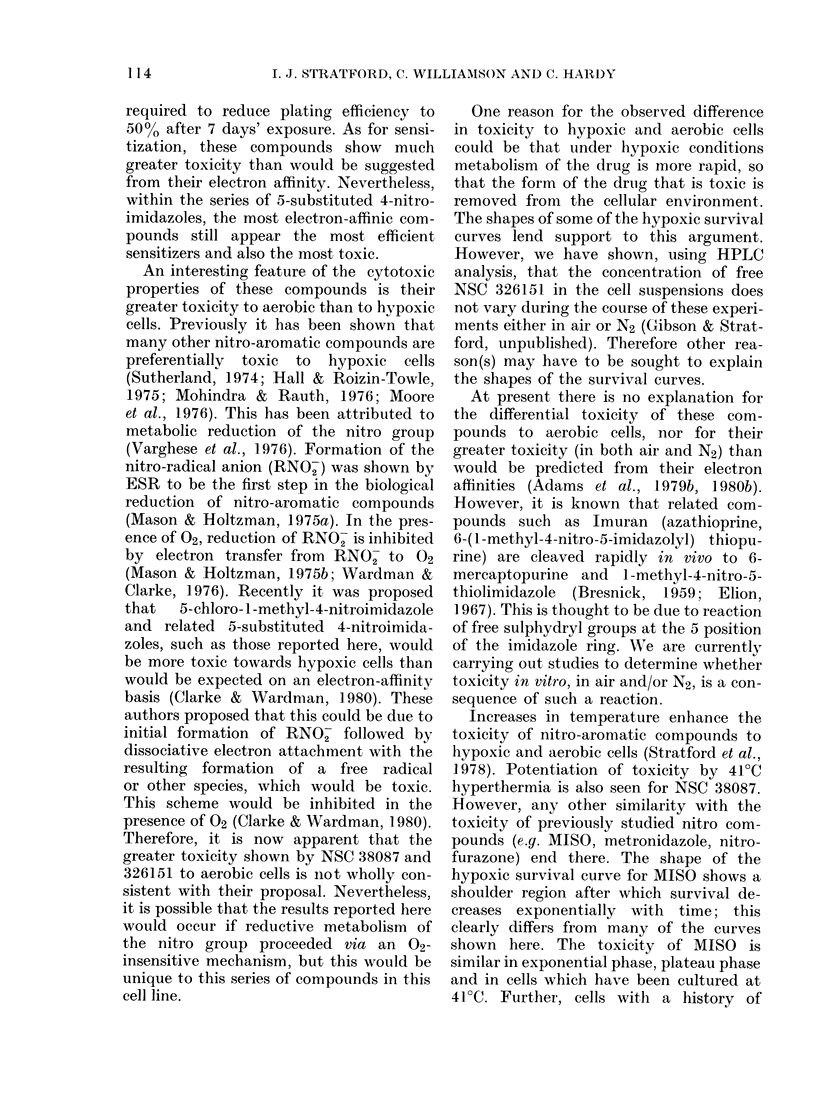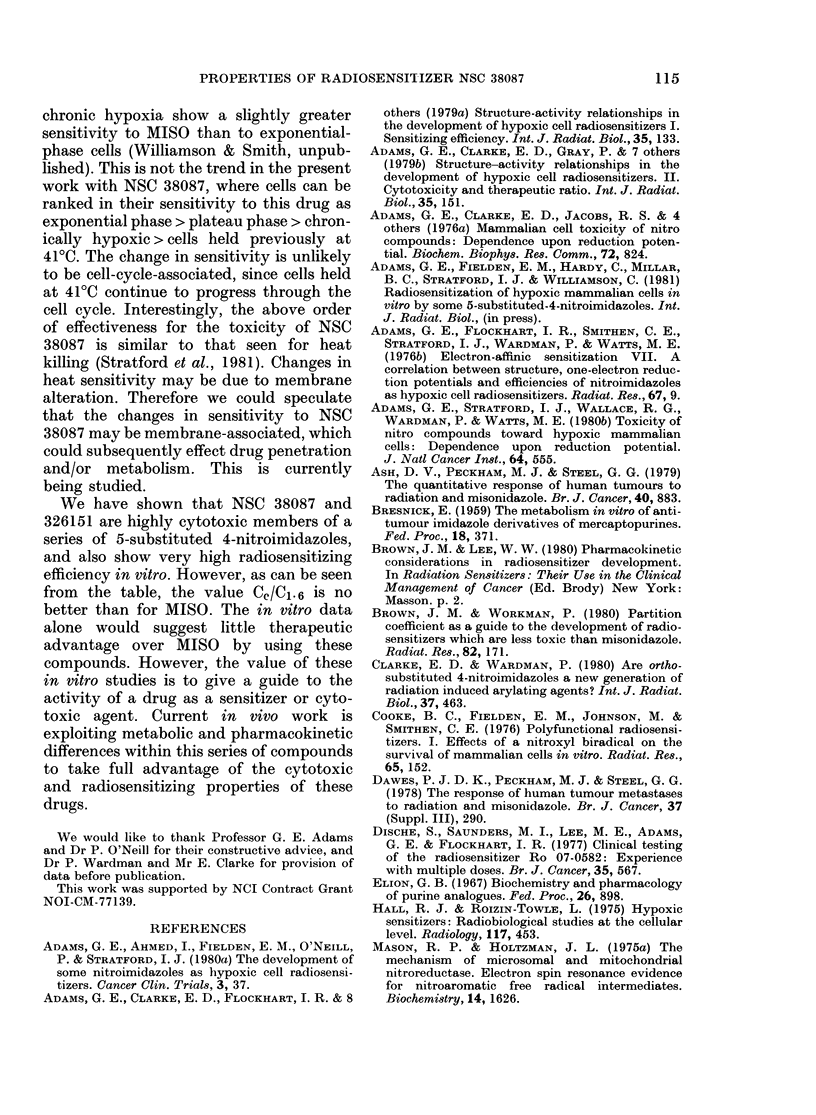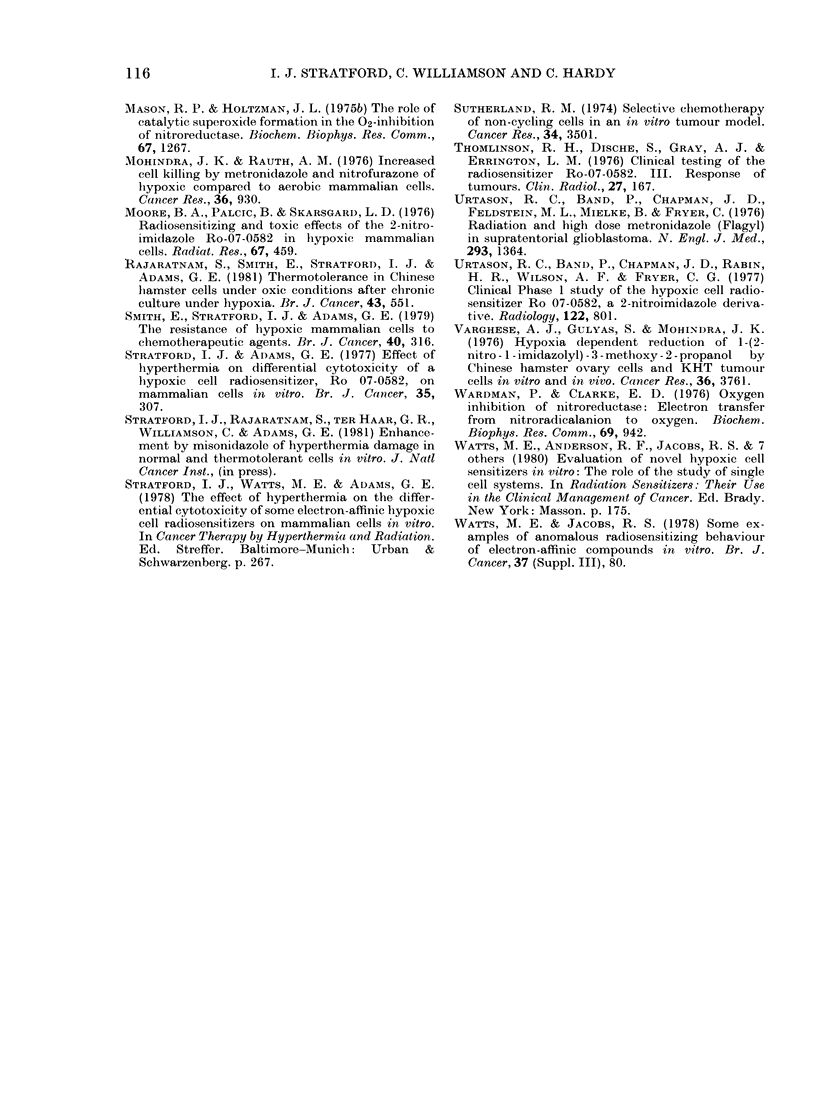# Cytotoxic properties of a 4-nitroimidazole (NSC 38087): a radiosensitizer of hypoxic cells in vitro.

**DOI:** 10.1038/bjc.1981.155

**Published:** 1981-07

**Authors:** I. J. Stratford, C. Williamson, C. Hardy

## Abstract

5-Phenoxysulphonyl-1-methyl-4-nitroimidazole (NSC 38087) can act as a sensitizer of hypoxic mammalian cells to radiation in vitro. The degree of sensitization achieved is greater than would be predicted from the drug's electron affinity. Cytotoxicity studies have shown that 5 microM NSC 38087 can reduce the surviving fraction of log-phase V79 cells in air at 37 degrees C to 10(-2) after 2 h exposure. This toxicity is considerably increased by small rises in temperature. In contrast to other nitro-heterocyclic radiosensitizers, NSC 38087 and related 5-substituted 4-nitroimidazoles show greater toxicity towards aerobic than to hypoxic cells. Log-phase cells show the highest sensitivity to the toxic action of NSC 38087, with plateau-phase cells, cells with a history of chronic hypoxia, and thermotolerant cells showing greater resistance. These toxicity data are compared to those for the 2-nitroimidazole hypoxic-cell sensitizer misonidazole.


*Br. J. Cancer* (1981) **44**, 109

### CYTOTOXIC PROPERTIES OF A 4-NITROIMIDAZOLE (NSC 38087): A RADIOSENSITIZER OF HYPOXIC CELLS *IN VITRO*

I. J. STRATFORD, C. WILLIAMSON AND C. HARDY

*From the Physics Department, Institute of Cancer Research, Sutton, Surrey SM2 5PX*

Received 14 November 1980 Accepted 18 March 1981

**Summary.—5-Phenoxysulphonyl-1-methyl-4-nitroimidazole (NSC 38087) can act as a sensitizer of hypoxic mammalian cells to radiation *in vitro*. The degree of sensitization achieved is greater than would be predicted from the drug's electron affinity. Cytotoxicity studies have shown that 5μM NSC 38087 can reduce the surviving fraction of log-phase V79 cells in air at 37°C to $10^{-2}$ after 2 h exposure. This toxicity is considerably increased by small rises in temperature. In contrast to other nitroheterocyclic radiosensitizers, NSC 38087 and related 5-substituted 4-nitroimidazoles show greater toxicity towards aerobic than to hypoxic cells. Log-phase cells show the highest sensitivity to the toxic action of NSC 38087, with plateau-phase cells, cells with a history of chronic hypoxia, and thermotolerant cells showing greater resistance. These toxicity data are compared to those for the 2-nitroimidazole hypoxic-cell sensitizer misonidazole.**

THE NITROIMIDAZOLES misonidazole (MISO) and metronidazole have been used in clinical trials as potential sensitizers of the radio-resistant hypoxic cells in tumours. In instances where clinical data have been evaluable some improvement in radiation response has been observed with these compounds (Urtason *et al.*, 1976; Thomlinson *et al.*, 1976; Dawes *et al.*, 1978; Ash *et al.*, 1979). However, these drugs show some neurotoxic effects which prevent their use at doses sufficient to give optimal sensitization (Dische *et al.*, 1977; Urtason *et al.*, 1977). This has led to a search for sensitizers more effective than MISO or metronidazole. Interest has centred on modifying the nitroimidazole compounds to produce sensitizers more efficient than MISO but with similar toxicity (Adams *et al.*, 1979*a*, *b*; Adams *et al.*, 1980*a*) or alternatively to produce a drug as good a sensitizer as MISO but with lower toxicity (Brown & Workman, 1980; Brown & Lee, 1980).

Using mammalian cells cultured *in vitro*, it has been shown that electron affinity is the predominant factor which determines both the cytotoxicity and sensitizing effectiveness of hypoxia-mediated drugs. There are, however, a number of exceptions to these general relationships which provide grounds for optimism that other parameters may be identified that can affect sensitization by and toxicity of nitroimidazoles. The compound 5-chloro-1-methyl-4-nitroimidazole has previously been shown to sensitize at concentrations about 100-fold lower than would have been predicted on the basis of electron affinity (Watts & Jacobs, 1978). In a subsequent study this anomalously high sensitizing efficiency was demonstrated for a range of 4-nitroimidazoles substituted in the 5-position with a sulphonate or sulphonamide group (Adams *et al.*, 1980*a*, 1981). Among these compounds, NSC 38087, with the structure shown in Fig. 1, which has an electron affinity only slightly greater than that of MISO, shows a sensitizing efficiency similar to that of $O_2$. In this paper we examine

FIG. 1.—Structure of NSC 38087; 5-phenoxy-sulphonyl-1-methyl-nitroimidazole.

the cytotoxic properties of NSC 38087, and its mode of action is compared to that of MISO.

### MATERIALS AND METHODS

*Cells.*—Chinese hamster V79-379A cells used in this work were maintained in spinner culture in Eagles' minimal essential medium (MEM) modified for suspension cultures (Flow Laboratories Ltd.) supplemented with 7·5% foetal calf serum (FCS, Gibco-Biocult Ltd.). The cells with a doubling time of ~10 h were kept in exponential phase at concentrations ranging between $10^5$ and $10^6$ cells per ml. For most experiments cells were harvested from asynchronous exponential-phase cultures. However, occasionally experiments were done on plateau-phase cells, cells with a history of chronic hypoxia and thermotolerant cells. Plateau-phase cells were prepared by seeding cultures at $10^5$/ml and holding in spinner culture at 37°C for 3 days before use. Cells with a history of chronic hypoxia were prepared by taking log-phase cells and holding them at pH 7·4 for 16 h under anaerobic conditions (Smith *et al.*, 1979; Rajaratnam *et al.*, 1981). Thermotolerant cells were prepared by holding log-phase cells for 16 h at 41°C; under which condition V79 cells are still capable of progression with a doubling time of 22–24 h. These cells are more resistant to subsequent hyperthermic damage than cells not receiving the 41°C treatment (Stratford *et al.*, 1981). All these cell populations had plating efficiencies ≥90% before cytotoxic treatment.

*Cytotoxicity experiments.*—250ml spinner flasks were fitted with a gas inlet/outlet system and a sidearm through which samples could be withdrawn. Cells at a concentration of $2 \times 10^5$ cells/ml were suspended in MEM + 7·5% FCS and held in a water bath at 37°C. The compounds were dissolved in MEM + 7·5% FCS and added to the suspension, which was buffered with bicarbonate to pH 7·4. When appropriate, the spinner containing cells was de-aerated by flowing $N_2$ plus 5% $CO_2$ (<10 pt/$10^6$ $O_2$; BOC Ltd.) at 500 ml per min over the surface of the stirred suspension throughout the experiment. Samples of cells were withdrawn at appropriate times, centrifuged, resuspended, counted and diluted. For each test condition various numbers of cells were plated, in triplicate, in MEM + 15% FCS and incubated for 7–10 days at 37°C before scoring for colony formation. Further details of the technique are described elsewhere (Stratford & Adams, 1977).

*Radiation experiments.*—Cells were harvested from exponential-phase cultures, diluted appropriately and added to glass Petri dishes containing 2·5 ml MEM, supplemented with 15% FCS. Cells were allowed to attach at 37°C for 1½ h before the medium was removed and replaced with fresh medium containing drug. Irradiations with $^{60}Co$ γ-rays were carried out in "dural" containers which can hold 4 Petri dishes (Cooke *et al.*, 1976). These vessels could be rendered hypoxic by purging with $N_2$ + 5% $CO_2$ (BOC Ltd.) for 1 h, when the vessels were sealed and irradiated at room temperature at a dose rate of 4·2 Gy/min. For irradiations in air, cells were equilibrated with air + 5% $CO_2$. After irradiation, the medium was removed and replaced with fresh MEM + 15% FCS and the cells incubated for 7–9 days before scoring for colony formation.

*Compounds.*—NSC 38087 was provided by the Drug Synghesis and Development Branch of NCI, U.S.A. NSC 326151 was synthesized in our laboratories.

### RESULTS

Fig. 2 illustrates the ability of NSC 38087 to radiosensitize hypoxic mammalian cells *in vitro*. There is no sensitization in air. However, under hypoxic conditions, 2μM NSC 38087 (0·52 μg/ml) gives an enhancement ratio, ER, of 1·5, as measured by the ratio of the slopes of the dose–log-survival curves. The inset to this figure shows a plot of ER *vs* concentration of NSC 38087, and it illustrates that this

FIG. 2.—Survival data for $\gamma$-irradiated V79 cells. △, $N_2$—no drug; ○, $N_2$—$2\mu$M NSC 38087; ▲, aerated cells—no drug; ●, aerated cells—$2\mu$M NSC 38087. The inset shows the dependence of enhancement ratio for irradiated cells on NSC 38087 concentration (data from Adams *et al.*, 1981).

compound is considerably more efficient as a sensitizer than MISO.

Not only does NSC 38087 show very high sensitizing efficiency, but it also shows considerable cytotoxicity towards V79 cells held under aerobic conditions at 37°C. Fig. 3 illustrates the toxicity of varying concentrations of NSC 38087 plotted as a function of time of exposure to drug. Data in this and other figures (unless indicated otherwise) come from individual experiments; replicate experiments were always done and provided similar results. It is apparent that the cytotoxicity of NSC 38087 is both concentration and time-dependent. However, there is an indication that, depending on the drug concentration, survival reaches a

FIG. 3.—Toxicity of NSC 38087 to aerobic Chinese hamster V79-379A cells at 37°C.

minimum level with longer exposures, producing no further cell killing (see, *e.g.*, data for $3{\cdot}5\mu$M NSC 38087). Further experiments showed that cells exposed to $3{\cdot}5\mu$M NSC 38087 for up to 23 h gave a similar surviving fraction to cells exposed

FIG. 4.—Survival of cells exposed to various concentrations of NSC 38087 for various times under aerobic conditions at 41°C. Data from Fig. 3 (37°C), generated in the same experiment as in this figure, has been transposed for comparison (– – – –).

FIG. 5.—Toxicity of 5$\mu$M NSC 38087 to hypoxic (○) and aerobic cells (△) at 37°C. Values ±s.e. from 6 experiments (air) and 4 ($N_2$).

FIG. 6.—Toxicity of 50$\mu$M NSC 326151 to hypoxic and aerobic cells at 37°C.

to the same concentration for only 5 h. The toxicity of NSC 38087 can be potentiated by small rises in temperature. Fig. 4 shows results for various concentrations of NSC 38087 in air at 41°C. This temperature alone causes no change in plating efficiency for V79 cells over the duration of these experiments; however, in the presence of NSC 38087 there is considerably more damage than that at 37°C. This is illustrated by the data for 3·5$\mu$M NSC 38087, where the 4° rise in temperature provides a 100-fold increase in cell killing after contact for 2 h or more.

A comparison of the toxicity of 5$\mu$M NSC 38087 towards hypoxic and aerobic cells is shown in Fig. 5. At this and other concentrations (data not shown) the nitroimidazole drug is more toxic to *aerobic* than hypoxic cells. This contrasts with all previous data for nitro-heterocyclic radiosensitizers, which have shown increased toxicity to *hypoxic* cells (Sutherland, 1974; Hall & Roizin-Towle, 1975; Mohindra & Rauth, 1976; Moore *et al.*, 1976). Other 5-substituted 4-nitroimidazoles which have sensitizing efficiencies much greater than would be expected from the electron affinities also show increased toxicity towards aerobic cells. Typical of such data are those given in Fig. 6 for 1-methyl-4-nitro-5-morpholino-sulphonylimidazole, NSC 326151. Interestingly, the concentration of NSC 326151 used in the above experiment is 10 times greater than for NSC 38087; yet similar amounts of cell killing are seen in both air and $N_2$. This may be due to the higher electron affinity of NSC 38087 (see Table).

All the previous experiments were

TABLE.—*Some radiosensitizing and cytotoxic properties of the 5-substituted 4-nitroimidazoles*†

| NSC code | $E_7^1$/mV | $C_{1\cdot6}$ (mmol/dm³) | $\hat{C}_{1\cdot6}$ (mmol/dm³) | $C_c$ (mmol/dm³) | $\hat{C}_c$ (mmol/dm³) | $(C_c/C_{1\cdot6})$* |
|---|---|---|---|---|---|---|
| 38087 | −342 | $2\cdot6\times10^{-3}$ | $1\cdot9\times10^{-1}$ | $9\cdot0\times10^{-4}$ | $1\cdot9\times10^{-1}$ | 0·35 |
| 326151 | −406 | $1\cdot5\times10^{-2}$ | $6\cdot2\times10^{-1}$ | $2\cdot0\times10^{-3}$ | $6\cdot6\times10^{-1}$ | 0·13 |

* For comparison, values of $C_c/C_{1\cdot6}$ for MISO range from 0·3 (Adams *et al.*, 1979*b*) to 1·0 (Watts *et al.*, 1980).
† $E_7^1$ is the one-electron reduction potential measured at pH 7·0; $C_{1\cdot6}$, $\hat{C}_{1\cdot6}$ are concentrations required to give a radiosensitizer enhancement ratio of 1·6; $C_c$ and $\hat{C}_c$ are concentrations required to reduce plating efficiency by 50% on incubation of cells with drug for 7–10 days. Data for values of $E_7^1$ and $C_{1\cdot6}$ are from Adams *et al.* (1980*a*, 1981). $\hat{C}_{1\cdot6}$ and $\hat{C}_c$ are values of sensitizing efficiency and chronic aerobic toxicity predicted from previous electron affinity correlations (Adams *et al.*, 1979*a*, *b*).

FIG. 7.—Survival of V79 cells with different cultural histories exposed to 5μM NSC 38087 under aerobic conditions at 37°C.

carried out on cells harvested from exponential-phase cultures. In order to determine the likely spectrum of effectiveness of these drugs, cytotoxicity experiments were done on V79 cells with different culture histories. Fig. 7 shows the effect of 5μM NSC 38087 on exponential, plateau and thermotolerant cells, and in addition on cells with a history of chronic hypoxia. All treatments were in air at 37°C. Clearly, the different cell populations respond to NSC 38087 in widely differing ways, with exponential cells showing the greatest, and thermotolerant cells the least sensitivity.

### DISCUSSION

NSC 38087 has the typical properties of a series of 5-sulphonyl derivatives of 1-methyl-4-nitroimidazole. These compounds show much greater sensitizing efficiency (Adams *et al.*, 1980*a*, 1981) and greater toxicity than would be suggested from their electron affinity. Some sensitization and toxicity data for NSC 38087 and NSC 326151, together with their one-electron reduction potentials ($E^1_7$) are recorded in the Table. Previously it has been shown for uncharged 5-nitrofurans, 2-nitro- and 5-nitroimidazoles, nitrobenzenes, quinones and some unsubstituted 4-nitroimidazoles that sensitizing efficiency and aerobic toxicity correlate extremely well with the electron affinity of the compounds (Adams *et al.*, 1976*a*, *b*, 1979*a*, *b*). Sensitizing efficiencies were defined as the concentration of drug required to achieve a given level of enhancement, usually 1·6 ($C_{1\cdot6}$). These values for the compounds used in the present work are given in the table, together with those values ($\hat{C}_{1\cdot6}$) which would have been predicted from previous electron-affinity correlations. There are orders-of-magnitude differences between the observed and predicted sensitizing efficiency. Values for the chronic aerobic toxicity of NSC 38087 and NSC 326151 are also given. These values are defined as the concentration of drug required to reduce plating efficiency to 50% after 7 days' exposure. As for sensitization, these compounds show much greater toxicity than would be suggested from their electron affinity. Nevertheless, within the series of 5-substituted 4-nitroimidazoles, the most electron-affinic compounds still appear the most efficient sensitizers and also the most toxic.

An interesting feature of the cytotoxic properties of these compounds is their greater toxicity to aerobic than to hypoxic cells. Previously it has been shown that many other nitro-aromatic compounds are preferentially toxic to hypoxic cells (Sutherland, 1974; Hall & Roizin-Towle, 1975; Mohindra & Rauth, 1976; Moore *et al.*, 1976). This has been attributed to metabolic reduction of the nitro group (Varghese *et al.*, 1976). Formation of the nitro-radical anion ($RNO_2^-$) was shown by ESR to be the first step in the biological reduction of nitro-aromatic compounds (Mason & Holtzman, 1975*a*). In the presence of $O_2$, reduction of $RNO_2^-$ is inhibited by electron transfer from $RNO_2^-$ to $O_2$ (Mason & Holtzman, 1975*b*; Wardman & Clarke, 1976). Recently it was proposed that 5-chloro-1-methyl-4-nitroimidazole and related 5-substituted 4-nitroimidazoles, such as those reported here, would be more toxic towards hypoxic cells than would be expected on an electron-affinity basis (Clarke & Wardman, 1980). These authors proposed that this could be due to initial formation of $RNO_2^-$ followed by dissociative electron attachment with the resulting formation of a free radical or other species, which would be toxic. This scheme would be inhibited in the presence of $O_2$ (Clarke & Wardman, 1980). Therefore, it is now apparent that the greater toxicity shown by NSC 38087 and 326151 to aerobic cells is not wholly consistent with their proposal. Nevertheless, it is possible that the results reported here would occur if reductive metabolism of the nitro group proceeded *via* an $O_2$-insensitive mechanism, but this would be unique to this series of compounds in this cell line.

One reason for the observed difference in toxicity to hypoxic and aerobic cells could be that under hypoxic conditions metabolism of the drug is more rapid, so that the form of the drug that is toxic is removed from the cellular environment. The shapes of some of the hypoxic survival curves lend support to this argument. However, we have shown, using HPLC analysis, that the concentration of free NSC 326151 in the cell suspensions does not vary during the course of these experiments either in air or $N_2$ (Gibson & Stratford, unpublished). Therefore other reason(s) may have to be sought to explain the shapes of the survival curves.

At present there is no explanation for the differential toxicity of these compounds to aerobic cells, nor for their greater toxicity (in both air and $N_2$) than would be predicted from their electron affinities (Adams *et al.*, 1979*b*, 1980*b*). However, it is known that related compounds such as Imuran (azathioprine, 6-(1-methyl-4-nitro-5-imidazolyl) thiopurine) are cleaved rapidly *in vivo* to 6-mercaptopurine and 1-methyl-4-nitro-5-thiolimidazole (Bresnick, 1959; Elion, 1967). This is thought to be due to reaction of free sulphydryl groups at the 5 position of the imidazole ring. We are currently carrying out studies to determine whether toxicity *in vitro*, in air and/or $N_2$, is a consequence of such a reaction.

Increases in temperature enhance the toxicity of nitro-aromatic compounds to hypoxic and aerobic cells (Stratford *et al.*, 1978). Potentiation of toxicity by 41°C hyperthermia is also seen for NSC 38087. However, any other similarity with the toxicity of previously studied nitro compounds (*e.g.* MISO, metronidazole, nitrofurazone) end there. The shape of the hypoxic survival curve for MISO shows a shoulder region after which survival decreases exponentially with time; this clearly differs from many of the curves shown here. The toxicity of MISO is similar in exponential phase, plateau phase and in cells which have been cultured at 41°C. Further, cells with a history of chronic hypoxia show a slightly greater sensitivity to MISO than to exponential-phase cells (Williamson & Smith, unpublished). This is not the trend in the present work with NSC 38087, where cells can be ranked in their sensitivity to this drug as exponential phase > plateau phase > chronically hypoxic > cells held previously at 41°C. The change in sensitivity is unlikely to be cell-cycle-associated, since cells held at 41°C continue to progress through the cell cycle. Interestingly, the above order of effectiveness for the toxicity of NSC 38087 is similar to that seen for heat killing (Stratford *et al.*, 1981). Changes in heat sensitivity may be due to membrane alteration. Therefore we could speculate that the changes in sensitivity to NSC 38087 may be membrane-associated, which could subsequently effect drug penetration and/or metabolism. This is currently being studied.

We have shown that NSC 38087 and 326151 are highly cytotoxic members of a series of 5-substituted 4-nitroimidazoles, and also show very high radiosensitizing efficiency *in vitro*. However, as can be seen from the table, the value $C_c/C_{1\cdot 6}$ is no better than for MISO. The *in vitro* data alone would suggest little therapeutic advantage over MISO by using these compounds. However, the value of these *in vitro* studies is to give a guide to the activity of a drug as a sensitizer or cytotoxic agent. Current *in vivo* work is exploiting metabolic and pharmacokinetic differences within this series of compounds to take full advantage of the cytotoxic and radiosensitizing properties of these drugs.

We would like to thank Professor G. E. Adams and Dr P. O'Neill for their constructive advice, and Dr P. Wardman and Mr E. Clarke for provision of data before publication.

This work was supported by NCI Contract Grant NOI-CM-77139.

### REFERENCES

Adams, G. E., Ahmed, I., Fielden, E. M., O'Neill, P. & Stratford, I. J. (1980*a*) The development of some nitroimidazoles as hypoxic cell radiosensitizers. *Cancer Clin. Trials*, **3**, 37.

Adams, G. E., Clarke, E. D., Flockhart, I. R. & 8 others (1979*a*) Structure-activity relationships in the development of hypoxic cell radiosensitizers I. Sensitizing efficiency. *Int. J. Radiat. Biol.*, **35**, 133.

Adams, G. E., Clarke, E. D., Gray, P. & 7 others (1979*b*) Structure–activity relationships in the development of hypoxic cell radiosensitizers. II. Cytotoxicity and therapeutic ratio. *Int. J. Radiat. Biol.*, **35**, 151.

Adams, G. E., Clarke, E. D., Jacobs, R. S. & 4 others (1976*a*) Mammalian cell toxicity of nitro compounds: Dependence upon reduction potential. *Biochem. Biophys. Res. Comm.*, **72**, 824.

Adams, G. E., Fielden, E. M., Hardy, C., Millar, B. C., Stratford, I. J. & Williamson, C. (1981) Radiosensitization of hypoxic mammalian cells *in vitro* by some 5-substituted-4-nitroimidazoles. *Int. J. Radiat. Biol.*, (in press).

Adams, G. E., Flockhart, I. R., Smithen, C. E., Stratford, I. J., Wardman, P. & Watts, M. E. (1976*b*) Electron-affinic sensitization VII. A correlation between structure, one-electron reduction potentials and efficiencies of nitroimidazoles as hypoxic cell radiosensitizers. *Radiat. Res.*, **67**, 9.

Adams, G. E., Stratford, I. J., Wallace, R. G., Wardman, P. & Watts, M. E. (1980*b*) Toxicity of nitro compounds toward hypoxic mammalian cells: Dependence upon reduction potential. *J. Natl Cancer Inst.*, **64**, 555.

Ash, D. V., Peckham, M. J. & Steel, G. G. (1979) The quantitative response of human tumours to radiation and misonidazole. *Br. J. Cancer*, **40**, 883.

Bresnick, E. (1959) The metabolism *in vitro* of antitumour imidazole derivatives of mercaptopurines. *Fed. Proc.*, **18**, 371.

Brown, J. M. & Lee, W. W. (1980) Pharmacokinetic considerations in radiosensitizer development. In *Radiation Sensitizers: Their Use in the Clinical Management of Cancer* (Ed. Brody) New York: Masson. p. 2.

Brown, J. M. & Workman, P. (1980) Partition coefficient as a guide to the development of radiosensitizers which are less toxic than misonidazole. *Radiat. Res.*, **82**, 171.

Clarke, E. D. & Wardman, P. (1980) Are *ortho*-substituted 4-nitroimidazoles a new generation of radiation induced arylating agents? *Int. J. Radiat. Biol.*, **37**, 463.

Cooke, B. C., Fielden, E. M., Johnson, M. & Smithen, C. E. (1976) Polyfunctional radiosensitizers. I. Effects of a nitroxyl biradical on the survival of mammalian cells *in vitro*. *Radiat. Res.*, **65**, 152.

Dawes, P. J. D. K., Peckham, M. J. & Steel, G. G. (1978) The response of human tumour metastases to radiation and misonidazole. *Br. J. Cancer*, **37** (Suppl. III), 290.

Dische, S., Saunders, M. I., Lee, M. E., Adams, G. E. & Flockhart, I. R. (1977) Clinical testing of the radiosensitizer Ro 07-0582: Experience with multiple doses. *Br. J. Cancer*, **35**, 567.

Elion, G. B. (1967) Biochemistry and pharmacology of purine analogues. *Fed. Proc.*, **26**, 898.

Hall, R. J. & Roizin-Towle, L. (1975) Hypoxic sensitizers: Radiobiological studies at the cellular level. *Radiology*, **117**, 453.

Mason, R. P. & Holtzman, J. L. (1975*a*) The mechanism of microsomal and mitochondrial nitroreductase. Electron spin resonance evidence for nitroaromatic free radical intermediates. *Biochemistry*, **14**, 1626.

MASON, R. P. & HOLTZMAN, J. L. (1975*b*) The role of catalytic superoxide formation in the $O_2$-inhibition of nitroreductase. *Biochem. Biophys. Res. Comm.*, **67**, 1267.

MOHINDRA, J. K. & RAUTH, A. M. (1976) Increased cell killing by metronidazole and nitrofurazone of hypoxic compared to aerobic mammalian cells. *Cancer Res.*, **36**, 930.

MOORE, B. A., PALCIC, B. & SKARSGARD, L. D. (1976) Radiosensitizing and toxic effects of the 2-nitroimidazole Ro-07-0582 in hypoxic mammalian cells. *Radiat. Res.*, **67**, 459.

RAJARATNAM, S., SMITH, E., STRATFORD, I. J. & ADAMS, G. E. (1981) Thermotolerance in Chinese hamster cells under oxic conditions after chronic culture under hypoxia. *Br. J. Cancer*, **43**, 551.

SMITH, E., STRATFORD, I. J. & ADAMS, G. E. (1979) The resistance of hypoxic mammalian cells to chemotherapeutic agents. *Br. J. Cancer*, **40**, 316.

STRATFORD, I. J. & ADAMS, G. E. (1977) Effect of hyperthermia on differential cytotoxicity of a hypoxic cell radiosensitizer, Ro 07-0582, on mammalian cells *in vitro*. *Br. J. Cancer*, **35**, 307.

STRATFORD, I. J., RAJARATNAM, S., TER HAAR, G. R., WILLIAMSON, C. & ADAMS, G. E. (1981) Enhancement by misonidazole of hyperthermia damage in normal and thermotolerant cells *in vitro*. *J. Natl Cancer Inst.*, (in press).

STRATFORD, I. J., WATTS, M. E. & ADAMS, G. E. (1978) The effect of hyperthermia on the differential cytotoxicity of some electron-affinic hypoxic cell radiosensitizers on mammalian cells *in vitro*. In *Cancer Therapy by Hyperthermia and Radiation*. Ed. Streffer. Baltimore–Munich: Urban & Schwarzenberg. p. 267.

SUTHERLAND, R. M. (1974) Selective chemotherapy of non-cycling cells in an *in vitro* tumour model. *Cancer Res.*, **34**, 3501.

THOMLINSON, R. H., DISCHE, S., GRAY, A. J. & ERRINGTON, L. M. (1976) Clinical testing of the radiosensitizer Ro-07-0582. III. Response of tumours. *Clin. Radiol.*, **27**, 167.

URTASON, R. C., BAND, P., CHAPMAN, J. D., FELDSTEIN, M. L., MIELKE, B. & FRYER, C. (1976) Radiation and high dose metronidazole (Flagyl) in supratentorial glioblastoma. *N. Engl. J. Med.*, **293**, 1364.

URTASON, R. C., BAND, P., CHAPMAN, J. D., RABIN, H. R., WILSON, A. F. & FRYER, C. G. (1977) Clinical Phase 1 study of the hypoxic cell radiosensitizer Ro 07-0582, a 2-nitroimidazole derivative. *Radiology*, **122**, 801.

VARGHESE, A. J., GULYAS, S. & MOHINDRA, J. K. (1976) Hypoxia dependent reduction of 1-(2-nitro-1-imidazolyl)-3-methoxy-2-propanol by Chinese hamster ovary cells and KHT tumour cells *in vitro* and *in vivo*. *Cancer Res.*, **36**, 3761.

WARDMAN, P. & CLARKE, E. D. (1976) Oxygen inhibition of nitroreductase: Electron transfer from nitroradicalanion to oxygen. *Biochem. Biophys. Res. Comm.*, **69**, 942.

WATTS, M. E., ANDERSON, R. F., JACOBS, R. S. & 7 others (1980) Evaluation of novel hypoxic cell sensitizers *in vitro*: The role of the study of single cell systems. In *Radiation Sensitizers: Their Use in the Clinical Management of Cancer*. Ed. Brady. New York: Masson. p. 175.

WATTS, M. E. & JACOBS, R. S. (1978) Some examples of anomalous radiosensitizing behaviour of electron-affinic compounds *in vitro*. *Br. J. Cancer*, **37** (Suppl. III), 80.